# Opioid-sparing effects of perioperative paracetamol and nonsteroidal anti-inflammatory drugs (NSAIDs) in children

**DOI:** 10.1111/pan.12163

**Published:** 2013-04-09

**Authors:** Ivan Wong, Celia St John-Green, Suellen M Walker

**Affiliations:** 1 Department of Anaesthesia, Great Ormond St Hospital for Children NHS Foundation TrustLondon, UK; 2 School of Clinical Medicine, University of Cambridge, Addenbrooke's HospitalCambridge, UK; 3 Portex Unit: Pain Research, UCL Institute of Child HealthLondon, UK

**Keywords:** pain, postoperative, child, analgesics, opioid, analgesics, non-narcotic, anti-inflammatory agents, nonsteroidal

## Abstract

**Background and Objectives:**

Perioperative pain in children can be effectively managed with systemic opioids, but addition of paracetamol or nonsteroidal anti-inflammatory drugs (NSAIDs) may reduce opioid requirements and potentially improve analgesia and/or reduce adverse effects.

**Methods:**

A systematic literature search was conducted to identify trials evaluating postoperative opioid requirements in children and comparing NSAID and/or paracetamol with placebo. Studies were stratified according to design: continuous availability of intravenous opioid (PCA/NCA) vs intermittent ‘as needed’ bolus; and single vs multiple dose paracetamol/NSAIDs. Primary outcome data were extracted, and the percentage decrease in mean opioid consumption was calculated for statistically significant reductions compared with placebo. Secondary outcomes included differences in pain intensity, adverse effects (sedation, respiratory depression, postoperative nausea and vomiting, pruritus, urinary retention, bleeding), and patient/parent satisfaction.

**Results:**

Thirty-one randomized controlled studies, with 48 active treatment arms compared with placebo, were included. Significant opioid sparing was reported in 38 of 48 active treatment arms, across 21 of the 31 studies. Benefit was most consistently reported when multiple doses of study drug were administered, and 24 h PCA or NCA opioid requirements were assessed. The proportion of positive studies was less with paracetamol, but was influenced by dose and route of administration. Despite availability of opioid for titration, a reduction in pain intensity by NSAIDs and/or paracetamol was reported in 16 of 29 studies. Evidence for clinically significant reductions in opioid-related adverse effects was less robust.

**Conclusion:**

This systematic review supports addition of NSAIDs and/or paracetamol to systemic opioid for perioperative pain management in children.

## Introduction

Systemic opioids are utilized for management of perioperative pain in children of all ages 1,2. As there are significant developmental changes in both the pharmacokinetic and pharmacodynamic profile of opioids, doses need to be adjusted according to age and weight and titrated against individual response to optimize analgesia and minimize adverse effects 3,4. This can be achieved by a range of systemic opioid delivery methods, including continuous background infusion, scheduled intermittent boluses 5, nurse-controlled analgesia (NCA) 2, or patient-controlled analgesia (PCA) 1.

Multi-modal analgesia is recommended for the management of pediatric perioperative pain 3,6,7 and has the potential to improve analgesic efficacy by simultaneously targeting different analgesic mechanisms and/or reducing the dose requirements of single agents, thereby minimizing dose-dependent adverse effects. Addition of nonsteroidal anti-inflammatory drugs (NSAIDs) and/or paracetamol (acetaminophen) to postoperative opioid regimes is well supported by analyses of adult data 8–11. The quantity and quality of evidence related to pediatric perioperative pain management continue to increase 3,12, and a recent meta-analysis reported a decrease in opioid dose requirements by perioperative NSAID administration in children 13.

Variations in design methodology and assessment tools can influence the sensitivity of pediatric analgesic clinical trials 14. This qualitative systematic review aims to stratify evidence according to study design and sensitivity and use within study comparisons to assess the degree to which addition of NSAIDs and/or paracetamol alters postoperative systemic opioid requirements in children. In addition, changes in secondary outcomes (pain scores, drug-related adverse effects, and patient or parental satisfaction) will be summarized.

## Methods

### Search strategy

Relevant studies were identified by searching electronic databases (PubMed, Embase, CINAHL, Cochrane Library, NHS Evidence) for randomized controlled trials (RCTs) evaluating combinations of systemic paracetamol and/or NSAIDs with systemic opioids for postoperative pain management in children. Key words were used to identify the ‘population’ (*Children, Pediatric, Pediatric, Neonate, Child, Newborn*), ‘intervention’ (*paracetamol OR acetaminophen OR NSAIDs OR individual drug names* AND *opioid OR individual opioid names),* and ‘outcomes’ (*opioid sparing, morbidity, pain score, sedation, respiratory depression, PONV, pruritus, urinary retention, and patient/parent satisfaction)*. Titles and abstracts up to January 2012 were included in the search. Additional relevant titles were identified by manual search of original articles, reviews, and related correspondence. Data were identified, extracted, and presented in accordance with Preferred Reporting Items for Systematic Reviews and Meta-Analyses (PRISMA) guidelines (www.prisma-statement.org).

### Selection criteria

The full reports of RCTs were retrieved and evaluated. Two authors (I.W. and C.St.JG.) independently assessed whether studies met the inclusion criteria, and all three authors discussed and resolved any discrepancies. Criteria for inclusion included the following:

1. Study type: double blind, placebo-controlled trials quantifying the effect of paracetamol and/or NSAID vs placebo on systemic opioid requirements
2. Participants: children (0–18 years) undergoing surgery under general anesthesia
3. Interventions: multiple or single doses of study drug (paracetamol or NSAID) were administered perioperatively (defined as the first dose administered within an hour prior to induction or following wound closure) by any systemic route (oral, rectal, intramuscular, or intravenous).

Studies were excluded if they had no control group, ongoing regional analgesia, or nonstandardized use of other analgesics that could confound the opioid dose requirements.

### Data extraction

Details of the study protocol were extracted and tabulated, including: age range of patients; number of patients in each treatment arm; type(s) and duration of surgery; dose regimes for study drugs and opioid (dose, frequency, timing, route and method of delivery); method of pain assessment; criteria for opioid administration; adverse effects; and duration of follow-up. Each included study was graded for quality and scored using the Jadad criteria 15. In addition, the retrieved reports were grouped according to the following aspects of study design:

1. Continuous availability of intravenous opioid titrated according to individual response by PCA, NCA, or variable rate continuous infusion
2. Intermittent as needed opioid bolus administration
3. Use of regular repeated doses of paracetamol/NSAIDs for at least 24 h
4. Use of single dose or less than 6 h paracetamol/NSAIDs.

Secondary outcome data were extracted and included measures of (i) potential opioid-related adverse effects (sedation, respiratory depression, postoperative, nausea and vomiting, pruritus, and urinary retention); (ii) NSAID (increased bleeding, renal dysfunction) and paracetamol (overdose/toxicity) adverse effects; (iii) pain scores; and (iv) patient and/or parent satisfaction.

### Analysis

The primary outcome was opioid dose requirement in the postoperative period. Studies are reported as ‘positive’ if a statistically significant reduction in opioid requirements was documented in pair-wise comparisons between the treatment (i.e., paracetamol and/or NSAID) and placebo arm, as previously used in an analysis of similar adult trials 16. The difference between the means of the treatment arms was expressed as a percentage of the corresponding value in the placebo group ([placebo—treatment/placebo] ×100). Treatment groups in which opioid consumption was not statistically significantly different from the placebo group were designated as ‘negative’ and assigned an opioid-sparing effect of zero. Due to variability in methodology and reporting, within study comparisons of secondary outcomes in treatment (NSAID or paracetamol) vs placebo groups are reported as being increased, decreased, or not different.

## Results

### Description and stratification of retrieved studies

**Figure 1:**
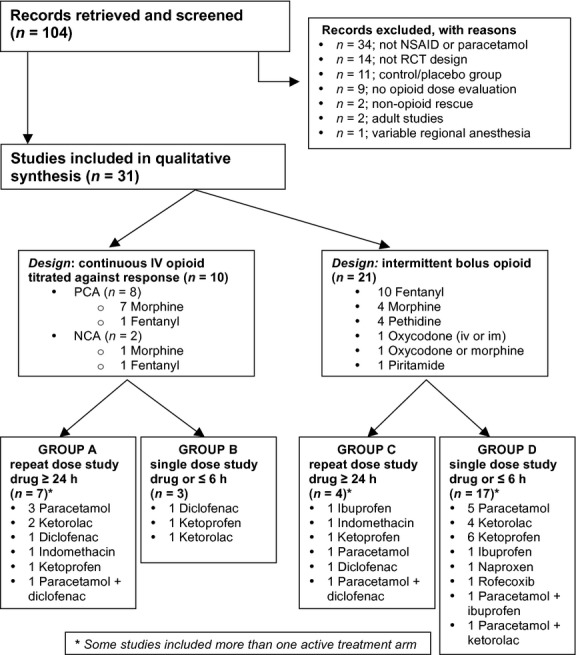
Flow chart of literature search with summary of excluded and included studies and grouping according to study design.

The systematic literature search yielded 104 relevant titles of which 31 met the inclusion criteria (Figure1). All included studies were placebo-controlled blinded trials with quality scores of 3–5 on the Jadad scale 15. Recruited children ranged in age from 1.5 months to 17 years old, and all received systemically administered opioids for perioperative analgesia. Several studies included multiple active treatment arms, but only those allowing comparison of opioid consumption in an active and placebo group were included. In total, 988 children were allocated to placebo control arms, and 1636 children received study drugs (paracetamol and/or NSAIDs). Numbers within treatment groups ranged from 13 to 84 subjects. Based on design methodology, studies were stratified according to the availability of opioid (continuous titration vs intermittent bolus) and the duration of study drug administration (either repeat dose for ≥24 h, or single dose ≤6 h) into four groups (Group A–D; Figure1).

In 10 of 31 studies, intravenous opioid was continuously available for titration (Group A and B; Table1). Morphine 17–24 or fentanyl 25,26 was administered via nurse-controlled (NCA; *n* = 2) or patient-controlled (PCA; *n* = 8) bolus administration. Lockout periods ranged from 10 to 30 min for NCA and 3 to 10 min for PCA, and a background infusion was included in six studies (four PCA and both NCA studies). In the 21 studies in Group C 27–30 and Group D 31–46 (Table2), opioid was available on an ‘as needed’ or ‘PRN’ basis, with nurse administration triggered by a predetermined pain score using either a formal assessment tool (16 studies) or global nursing assessment and patient request (five studies)(Table3).

**Table 1:** Study characteristics group A and B: interventions, analgesic administration, and opioid-sparing effects

Author (reference no.)	Surgery and duration (mean ± sd of control group unless stated)	Intervention drug, route, and age (mean ± sd unless stated)	Timing, frequency, and dose of study drug administration	Control and age (mean ± sd unless stated)	Opioid	Timing, frequency, and dose of opioid	Reported opioid dose-sparing effect (% reduction)
GROUP A: Titration of systemic opioid (PCA or NCA) and study drug for ≥24 h							
Hong *et al*. 26	Ureteroneocystostomy Range: 65–135 mins	Paracetamol IV (*n *=* *31) Range: 6–24 months	LOADING: 15 mg·kg^−1^ at end of surgery BOLUS (in NCA pump): 1.5 mg·kg^−1^ with 30 min lockout INFUSION: 1.5 mg·kg^−1^·h^−1^ for 72 h 6 h maximum: 15 mg·kg^−1^ paracetamol	Saline (*n *=* *32) Range:6–22 months	Fentanyl IV-NCA or parent controlled analgesia with background	LOADING DOSE: 0.5 mcg·kg^−1^ at end of surgery BACKGROUND: 0.25 mcg·kg^−1^·h^−1^ for 72 h BOLUS: 0.25 mcg·kg^−1^ with 30 min lockout TRIGGER: CHEOPS >4 4–13	POSITIVE* (54%) Paracetamol – 8.3 ± 3.7 Control – 18.1 ± 4.6 mcg·kg^−1^·day^−1^, total dose in first 24 h post-op, mean ± sd POSITIVE* (57%) Paracetamol – 20 Control – 43 mcg·kg^−1^, cumulative fentanyl dose first 3 days, mean
Rugyte *et al*. 18	Thoracic pectus correction Range: 90–225 mins	Ketoprofen IV (*n *=* *14) Range: 13–15 years	LOADING: 1 mg·kg^−1^ at end of surgery MAINTENANCE: 1 mg·kg^−1^ at 8 h and 16 h	Saline (*n *=* *17) Range: 10–15 years	Morphine IV-PCA with background	LOADING DOSE: 40 mc·kg^−1^ BOLUS: 20 mcg·kg^−1^ with 5 min lockout BACKGROUND: 5 mcg·kg^−1^·h^−1^ TRIGGER: patient	POSITIVE* (27%) Ketoprofen – 490 ± 240 Control – 670 ± 200 mcg·kg^−1^ total dose in first 24 h post-op, mean ± sd
Van der Marel *et al*. 17	Major abdominal or thoracic (noncardiac) surgery Range: 60–365 mins	Paracetamol PR (*n *=* *29) Range: 0–9 months	LOADING: 30–40 mg·kg^−1^ at induction MAINTENANCE: 20 mg·kg^−1^6–8 hrly for 48 h	Placebo (*n *=* *25) Range: 0–10 months	Morphine IV-NCA with background	LOADING DOSE: 100 mcg·kg^−1^ at end of surgery BACKGROUND: 5–30 mcg·kg^−1^·h^−1^ (depending on gestational age and bolus requirement) BOLUS: 5 mcg·kg^−1^ with 10 min lockout TRIGGER: VAS ≥4 (0–10)	NEGATIVE (+10) Paracetamol −7.91 (6.59–14.02) Control – 7.19 (5.45–12.06) mcg·kg^−1^·h^−1^, total dose in first 48 h post-op, median (25–75th percentile)
Munro *et al*. 19	Posterior spinal fusion 331 ± 62 mins	Ketorolac IV (*n *=* *20) 13.9 ± 1.3 years	LOADING: 0.5 mg·kg^−1^ at end of surgery MAINTENANCE: 0.5 mg·kg^−1^ every 6 h for 36 h	Saline (*n *=* *15) 14.1 ± 1.2 years	Morphine IV-PCA with background	LOADING DOSE: 50–100 mcg·kg^−1^ BOLUS: 20 mcg·kg^−1^ (lockout not stated) BACKGROUND: 10 mcg·kg^−1^·h^−1^ TRIGGER: patient	EQUIVOCAL POSITIVE* (33%) Ketorolac – 0.06 ± 0.3 Control – 0.09 ± 0.05 mcg·kg^−1^·day^−1^ during PACU, mean ± sd POSITIVE* (30%) Ketorolac – 0.7 ± 0.4 Control – 1 ± 0.5 mcg·kg^−1^·day^−1^ post-op day 2, mean ± sd NEGATIVE (11% n.s.) Ketorolac – 0.8 ± 0.3 Control – 0.9 ± 0.4 mcg·kg^−1^·day^−1^ on post-op day 1
Morton *et al*. 20	Open Appendicectomy Duration not reported	Paracetamol PR (*n *=* *20) Range: 5–12 years Diclofenac PR (*n *=* *20) Range: 6–13 years Combination (*n *=* *20) Range: 7–13 years	LOADING: paracetamol 20 mg·kg^−1^ AND/OR diclofenac 1 mg·kg^−1^ MAINTENANCE: paracetamol 15 mg·kg^−1^ 6 hrly AND/OR diclofenac 1 mg·kg^−1^ 8 hrly for 24 h post-op	Control: no treatment (*n *=* *20) Range: 5–13 years	Morphine IV-PCA with background	BOLUS: 20 mcg·kg^−1^ with 5 min lockout BACKGROUND: 4 mcg·kg^−1^·h^−1^ infusion for first 12 h post-op TRIGGER: patient	POSITIVE* (Diclofenac 43%, Combination 36%) Diclofenac – 435 (101–1054) Combination – 484 (126–1214) Control – 759 (368–1914) NEGATIVE (Paracetamol 17% n.s) Paracetamol – 627 (124–1339) mcg·kg^−1^, total dose in first 24 h post-op, median (range)
Sutters *et al*. 22	Orthopedic surgery 178.2 ± 105.8 mins	Ketorolac IV (*n *=* *36) 12.6 ± 3.5 years	LOADING: 1 mg·kg^−1^ in recovery MAINTENANCE: 0.5 mg·kg^−1^ 6 hrly for 48 h post-op	Saline (*n *=* *32) 12.6 ± 4.2 years	Morphine IV-PCA	LOADING DOSE: NCA boluses in recovery BOLUS: 16 mcg·kg^−1^ with 10 min lockout TRIGGER: patient	POSITIVE* (47%) Ketorolac – 3.37 ± 2.66 Control – 6.38 ± 4.36 mg, total dose in first 24 h post-op, mean ± sd
Sims *et al*. 21	Emergency open appendicectomy 39 ± 12 mins	Indomethacin PR (*n *=* *13) 10.1 ± 1.8 years	LOADING: 2 mg·kg^−1^ at end of surgery MAINTENANCE: 2 mg·kg^−1^ at 12 h and 24 h post-op	Placebo (*n *=* *15) 10.7 ± 2.1 years	Morphine IV-PCA	BOLUS: 20 mcg·kg^−1^ with 3 min lockout TRIGGER: patient	POSITIVE* (44%) Indomethacin – 0.51 ± 0.34 Placebo – 0.91 ± 0.46 mg, total dose first 36 h post-op, mean ± sd
GROUP B: Titration of systemic opioid and single dose (≤6 h) study drug							
Antila *et al*. 25	Tonsillectomy 17 ± 7 mins	Ketoprofen IV (*n *=* *15) 12.5 ± 2.3 years	2 mg·kg^−1^ at induction PLUS 2 mg·kg^−1^ infusion over 6 h post-op	Saline (*n *=* *15) 12.5 ± 1.9 years	Fentanyl IV-PCA	BOLUS: 0.5 mcg·kg^−1^ with 5 min lockout TRIGGER: patient	POSITIVE* (14%) Ketoprofen – 11.9 ± 8.8 Placebo – 13.9 ± 7.9 mcg·kg^−1^ total dose in first 24 h post-op, mean ± sd
Oztekin *et al*. 24	Tonsillectomy ± adenoidectomy 57.8 ± 3.23 mins	Diclofenac PR (*n *=* *20) 8.40 ± 0.53 years	1 mg·kg^−1^ prior to incision	Control: no treatment (*n *=* *20) 8.90 ± 0.45 years	Morphine IV-PCA with background	LOADING DOSE: 50 mcg·kg^−1^ at end of surgery BOLUS: 20 mcg·kg^−1^ bolus with 5 min lockout BACKGROUND: 4 mcg·kg^−1^·h^−1^ infusion TRIGGER: patient	POSITIVE* (24%) Diclofenac – 130 ± 11.3 Control – 170 ± 9.22 microgram·kg^−1^, in PACU, mean ± sd
Vetter *et al*. 23	Orthopedic surgery (osteotomy, arthrodesis, ORIF) 113 ± 42 mins	Ketorolac IV (*n *=* *25) 13 ± 2.0 years	0.8 mg·kg^−1^ at end of surgery	Control: no treatment (*n *=* *25) 13 ± 2.0 years	Morphine IV-PCA	LOADING DOSE: 0.05 to 0.3 mg·kg^−1^ in recovery BOLUS: 15 mcg·kg^−1^ with 10 min lockout TRIGGER: patient	POSITIVE* (35%) Ketorolac – 0.017 ± 0.008 Control – 0.026 ± 0.011 mg·kg^−1^·h^−1^, in first 12 h post-op, mean ± sd

* Positive, statistically significant opioid dose-sparing effect reported by authors; Negative, no statistically significant difference in opioid requirements.

PR, per rectum; IV, intravenous; IM, intramuscular.

**Table 2:** Study characteristics group C and D: interventions, analgesic administration and opioid-sparing effects

Author (reference no.)	Surgery and duration (mean ± sd of control group unless stated)	Intervention drug, route and age (mean ± sd unless stated)	Timing, frequency and dose of study drug administration	Control and age (mean ± sd unless stated)	Opioid	Frequency and dose of opioid trigger (pain score) for administration	Opioid dose-sparing effect (% reduction)
GROUP C: Intermittent opioid bolus and study drug for ≥24 h							
Mireskandari 27	Cleft palate repair 81.7 ± 30.1 mins	Paracetamol PR (*n *=* *30) 2.1 ± 0.8 years Diclofenac PR (n = 30) 2.2 ± 1.1 years Combination (*n *=* *30) 2.0 ± 0.5 years	30 mg·kg^−1^ paracetamol, 1 mg·kg^−1^ diclofenac or combination 8 hrly for 48 h	Placebo PR (*n *=* *30) 2.3 ± 1 year	Pethidine IM	BOLUS: 1 mg·kg^−1^ IM TRIGGER: CHEOPS >7	POSITIVE* (9 – 47%) Paracetamol – 88.3 ± 8.3 (9%) Diclofenac – 70.7 ± 10 (27%) Combined – 51 ± 9.5 (47%) Control – 97.1 ± 13.9 Total pethidine dose in first 48 h post-op Paracetamol – 6.2 ± 0.6 (11%) Diclofenac – 4.9 ± 0.7 (28%) Combined – 3.6 ± 0.7 (47%) Control – 6.8 ± 0.9 No. of doses in first 48 h
Kokki *et al*. 28	Tonsillectomy 25 ± 12 mins	Ketoprofen IV Pre-op (*n *=* *47) 10 ± 1 years post-op (n = 42) 12 ± 3 years	LOADING: 0.5 mg·kg^−1^ after induction (n = 47) OR end of surgery (*n *=* *42) MAINTENANCE: 3 mg·kg^−1^ infusion over 24 h	Saline (*n *=* *20) 11 ± 1 years	Oxycodone IV or IM	BOLUS: 0.05 mg·kg^−1^ IV or 0.1 mg·kg^−1^ IM TRIGGER: VAS >30 mm	NEGATIVE Preop ketoprofen – 4.1 ± 2.7 (19% n.s.) post-op ketoprofen – 3.7 ± 2.4 (27% n.s.) Control – 5.1 ± 2.7 No. of doses in first 24 h post-op, mean ± sd
Maunuksela *et al*. 29	Ophthalmic, general or orthopedic surgery Control group: 50.1 ± 39.4 mins	Ibuprofen PR (*n *=* *64) 7.7 ± 2.7 years	LOADING: 10 mg·kg^−1^ MAINTENANCE: total 40 mg·kg^−1^ 6–8 hrly for 24 h	Placebo z(*n *=* *64) 7.4 ± 2.7 years	Morphine or Oxycodone (different centers) IV or IM	BOLUS: 0.1 mg·kg^−1^ IV in recovery; 0.15 mg·kg^−1^ IM on ward TRIGGER: OPS moderate/severe or patient request	EQUIVOCAL POSITIVE* (orthop surgery 36%) Ibuprofen (n = 12) – 0.27 ± 0.20 Control (n = 19) – 0.42 ± 0.23 NEGATIVE (other surgery 25% n.s.) Ibuprofen (n = 52) – 0.09 ± 0.11 Control (n = 45) – 0.12 ± 0.1 mg·kg^−1^, total opioid dose in first 24 h post-op, mean ± sd
Maunuksela *et al*. 30	Orthopedic or general surgery 0.85 ± 0.61 h	Indomethacin IV (*n *=* *51) 7.0 ± 5.1 years	BOLUS: 0.35 mg·kg^−1^ at end of surgery MAINTENANCE: 0.07 mg·kg^−1^·h^−1^ for 24 h	Placebo (*n *=* *49) 6.1 ± 4.3 years	Morphine IV or IM	BOLUS: 0.1 mg·kg^−1^ IV in recovery; 0.15 mg·kg^−1^ IM on ward TRIGGER: Maunuksela score >3 or patient request	POSITIVE* (28%) Indomethacin – 0.24 ± 0.17 Control – 0.33 ± 0.21 mg·kg^−1^, total morphine dose in first 24 h post-op, mean ± sd
GROUP D: Intermittent opioid bolus and Single dose study drug							
Hong *et al*. 31	Inguinal hernia repair (day case) 33.0 ± 11.7 mins	Ketorolac + Paracetamol IV (*n *=* *28) 28.4 ± 15.5 months	1 mg·kg^−1^ ketorolac + 20 mg·kg^−1^ paracetamol after induction	Saline (*n *=* *27) 28.0 ± 13.3 months	Fentany lIV	BOLUS: 0.5 mcg·kg^−1^ TRIGGER: Wong-Baker score >2	POSITIVE* (61%) Ketorolac-Paracetamol – 0.54 ± 0.3 Control – 1.37 ± 0.2 microgram·kg^−1^, in PACU, mean ± sd
Dashti *et al*. 32	Adenotonsillectomy Duration not reported	Paracetamol PR (*n *=* *53) 10.2 ± 2.84 years	40 mg·kg^−1^ given after Induction	Control: no treatment (*n *=* *51) 9.45 ± 2.22 years	Pethidine (meperidine) IV	BOLUS: 0.5 mg·kg^−1^ TRIGGER: VAS>40 mm	POSITIVE* (62%) Paracetamol – 6.48 ± 8.52 Control - 17.09 ± 12.12 mg, total dose in first 24 h post-op, mean ± sd
Korpela *et al*. 33	Adenoidectomy (day case) Duration not reported	Paracetamol oral (*n *=* *30) Median & range: 1.7 (0.8 – 5) years Naproxen oral (*n *=* *30) median & range: 1.9 (0.8 – 7.8) years	Paracetamol 20 mg·kg^−1^ or Naproxen 10 mg·kg^−1^ given 0.5 h before induction	Placebo (*n *=* *30) median and range: 1.7 (0.8 – 6.2) years	Fentany lIV	BOLUS: 10 mcg·kg^−1^ TRIGGER: OPS >4	EQUIVOCAL POSITIVE* (Naproxen 19%) Naproxen – 17/30 (19%) Paracetamol – 20/30 (5% n.s.) Control – 21/30 Proportion requiring fentanyl in first 2 h post-op *Note: data only from groups given standardized intra-operative analgesia (pethidine 1 *mg·kg^−1^*)*
Sheeran *et al*. 34	Adenotonsillectomy (day case) Duration not reported	Rofecoxib oral (*n *=* *23) 7.2 ± 1.8 years	1 mg.kg given 0.5 h before induction	Placebo (*n *=* *22) 7.6 ± 2.2 years	Morphine IV	BOLUS: 25 mcg·kg^−1^ TRIGGER: nurse assessment	NEGATIVE (0%) Rofecoxib – 39 ± 28 Control – 39 ± 32 microgram·kg^−1^, in first 24 h post-op, mean ± sd
Viitanen *et al*. 35	Adenoidectomy (day case) 30 ± 9 mins	Paracetamol PR (*n *=* *40), 1.0–6.4 years Ibuprofen PR (*n *=* *40), 1.0–6.9 years Combination (*n *=* *40) 1.0–6.9 years	40 mg·kg^−1^ paracetamol; 15 mg·kg^−1^ ibuprofen; combination given at induction	Placebo (*n *=* *40) Range: 1.0–6.3 years	Pethidine (meperidine) IV	BOLUS: 5 or 10 mg TRIGGER: OPS >3	POSITIVE* (Paracetamol 19%, Ibuprofen 27%, Combination 28%) Paracetamo– 0.87 ± 0.39 (19%) Ibuprofen - 0.78 ± 0.37 (27%) Combination – 0.77 ± 0.45 (28%) Control – 1.07 ± 0.38 mg·kg^−1^, total dose in PACU, mean ± sd
Tuomilehto *et al*. 36	Adenoidectomy Duration not reported	Ketoprofen IV (*n *=* *40) Median: 30 (15–75) months Ketoprofen IM (*n *=* *40) Median: 42 (12–100) months	2 mg·kg^−1^ IV at induction OR 2 mg·kg^−1^ IM at induction	Saline (*n *=* *40) Median: 33 (16–85) months	Fentanyl IV	BOLUS: 0.5 mcg·kg^−1^ TRIGGER: Maunuksela >3	POSITIVE* (IV 28% IM 23%) IV - 25/40 IM - 27/40 Placebo - 35/40 Proportion requiring rescue analgesia in PACU
Bremerich *et al*. 37	Cleft palate repair Control group 109.9 ± 29.1 mins	Paracetamol PR mg·kg^−1^ (*n *=* *20) 11.7 ± 8.8 months 20 mg·kg^−1^ (*n *=* *20) 12.1 ± 10.4 months 40 mg·kg^−1^ (*n *=* *20) 9.5 ± 9.0 months	10 mg·kg^−1^ (*n *=* *20), 20 mg·kg^−1^ (*n *=* *20), or 40 mg·kg^−1^ (*n *=* *20), given at induction	Placebo (*n *=* *20) 12.5 ± 11.7 months	Piritramide IV	BOLUS: 25 mcg·kg^−1^ TRIGGER: CHIPPS >4	NEGATIVE Paracetamol 10 mg·kg^−1^ – 3.0 ± 1.2 (+10%) Paracetamol 20 mg·kg^−1^ – 3.2 ± 1.3 (+18%) Paracetamol 40 mg·kg^−1^ – 3.4 ± 1.1 (+26%) Placebo – 2.7 ± 1.6 Total no. of doses of piritramide in PACU, mean ± sd
Kokki *et al*. 38	Adenoidectomy Duration not reported	Ketoprofen PR (*n *=* *42) Median 33 (17–90) months Ketoprofen IV (*n *=* *42) Median: 44 (15–97) months	25 mg PR 30 mins prior to induction Or 25 mg IV at induction	Placebo PR & saline IV (*n *=* *39) Median: 45 (14–73) months	Fentanyl IV	BOLUS: 0.5 mcg·kg^−1^ in recovery TRIGGER: Maunuksela >3	POSITIVE*(PR 24% IV 22%) PR – 27/42 IV – 28/42 Control - 33/39 Proportion requiring rescue analgesia in PACU POSITIVE*(PR 52% IV 63%) PR – 9/42 IV – 7/42 Control – 17/39 Proportion requiring ≥3 rescue doses in PACU
Tuomilehto *et al*. 47	Adenoidectomy Duration: 17 8–37 mins	Ketoprofen PO (*n *=* *40) Median: 50 (20–101) months Ketoprofen IV (*n *=* *40) Median: 32 (16–82) months	1 mg·kg^−1^ PO 30 mins prior to induction Or 1 mg·kg^−1^ IV at induction	Saline PO & IV (*n *=* *20) Median: 46 (15–101) months	Fentanyl IV	BOLUS: 0.5 mcg·kg^−1^ in recovery TRIGGER: Maunuksela >3	EQUIVOCAL NEGATIVE IV – 30/40 Oral – 28/40 Control – 15/20 Proportion requiring rescue analgesia in PACU POSITIVE*Oral – 6/40 IV – 3/40 Control – 6/20 Proportion requiring ≥3 rescue doses in PACU
Kokki *et al*. 40	Strabismus surgery Median (quartiles): 30 23–45 mins	Ketoprofen IV (*n *=* *30) Median (quartiles): 83 (50–112) months	LOADING: 1 mg·kg^−1^ after induction MAINTENANCE: 1 mg·kg^−1^ infusion over 2 h	Saline (*n *=* *29) Median (quartiles): 64 (46–94) months	Fentany lIV	BOLUS: 1.0 mcg·kg^−1^ TRIGGER: Maunuksela >3	POSITIVE* (28%) Ketoprofen – 21/30 Control – 26/29 Proportion requiring fentanyl in first 2 h POSITIVE* (29%) Ketoprofen – 44 Control – 62 Total doses fentanyl per group first 2 h post-op
Korpela *et al*. 39	Inguinal surgery, adenoidectomy, general surgery (day case) Duration not reported	Paracetamol PR (*n *=* *90) 20 mg·kg^−1^ range: 1.0–7.2 years 40 mg·kg^−1^ range: 1.0–7.2 years 60 mg·kg^−1^ range: 1.1–7.8 years	20 mg·kg^−1^ (*n *=* *30), 40 mg·kg^−1^ (*n *=* *30), or 60 mg·kg^−1^ (*n *=* *30) given at induction	Placebo (*n *=* *30) 1.1–7.7 years	Morphine IV	BOLUS: 0.1 mg·kg^−1^ TRIGGER: nurse assessment	POSITIVE* (20 mg 27%, 40 mg 54%, 60 mg 73%) Paracetamol 20 mg·kg^−1^ – 0.8 Paracetamol 40 mg·kg^−1^ – 0.5 Paracetamol 60 mg·kg^−1^ – 0.3 Control – 1.1 Total no. of doses of morphine per patient in first 2 h post-op, mean
Kokki *et al*. 41	Adenoidectomy (day case) Duration not reported	Ketoprofen IV (*n *=* *165) 0.3 mg·kg^−1^ median (10th–90th percentile): 40 (18–74) months1.0 mg·kg^−1^: 32 (15–85) months3.0 mg·kg^−1^: 32 (15–72) months	0.3 mg·kg^−1^ (*n *=* *55) 1.0 mg·kg^−1^ (*n *=* *55) 3.0 mg·kg^−1^ (*n *=* *55) given at induction	Saline (*n *=* *55) Median (10th–90th percentile): 41 (15–79) months	FentanylI V	BOLUS: 1.0 mcg·kg^−1^ TRIGGER: Maunuksela >3	POSITIVE* (0.3 mg 21%, 1.0 mg 24%, 3.0 mg 35%) Ketoprofen 0.3 mg·kg^−1^ – 65% Ketoprofen 1.0 mg·kg^−1^ – 62% Ketoprofen 3.0 mg·kg^−1^ – 53% Control – 82% Proportion requiring fentanyl in first 2 h post-op
Romsing *et al*. 42	Tonsillectomy ±adenoidectomy Duration: 61.6 ± 22.0 mins	Ketorolac IV (*n *=* *40) Pre-op group: 9.4 ± 3.2 years post-op group: 9.7 ± 3.7 years	1.0 mg·kg^−1^ at induction (*n *=* *20) 1.0 mg·kg^−1^ at end of surgery (*n *=* *20)	Saline (*n *=* *20) 8.8 ± 3.2 years	FentanylI V	BOLUS: 0.5 or 1.0 mcg·kg^−1^ TRIGGER: nurse assessment or patient request	EQUIVOCAL (Pre-op 9%*, post-op 0%) Pre-op Ketorolac – 2/55 post-op Ketorolac – 9/55 Control – 7/55 Proportion requiring fentanyl in first 1.5 h post-op
Nikane *et al*. 43	Adenoidectomy ± myringotomy or tympanostomyDuration: not reported	Ketoprofen IV (*n *=* *80) 38 (12–111) months	1 mg·kg^−1^ bolus + 1 mg·kg^−1^ over 2 h	Saline (*n *=* *84) 40 (10–95) months	Fentanyl IV	BOLUS: 1 mcg·kg^−1^ TRIGGER: Maunuksela >3	POSITIVE* (13%) Ketoprofen – 51/80 – 64% Control – 65/84 – 77% Proportion requiring fentanyl in PACU
Bean-Lijewski *et al*. 44	Ilioinguinal or general surgery (day case) Duration: 93.0 ± 21.2 mins	Ketorolac IV (*n *=* *29) 4.0 ± 2.2 years	0.75 mg·kg^−1^ at induction	Saline (*n *=* *28) 4.2 ± 3.0 years	Pethidine (meperidine) IV	BOLUS: 0.5 mg·kg^−1^ TRIGGER: nurse assessment or patient request	POSITIVE* (46%) Ketorolac – 13/29 Control – 26/28 Proportion requiring pethidine in first 1 h post-op
Sutters *et al*. 45	Tonsillectomy (day case) Duration not reported	Ketorolac IM (*n *=* *45) 7.1 ± 2.4 years	1.0 mg·kg^−1^ at end of surgery	Saline (*n *=* *42) 7.1 ± 2.2 years	FentanylI V	BOLUS: 0.5 mcg·kg^−1^ TRIGGER: nurse assessment in recovery	POSITIVE* (26%) Ketorolac – 35.9 ± 2.5 Control – 48.3 ± 5.0 Total dose microgram per group until day stay discharge, mean ± sd
Watcha *et al*. 46	Adenotonsillectomy, moderate orthopedic or plastic surgery Duration: 87 ± 50 mins	Ketorolac IV (*n *=* *32) 8.3 ± 3.8 years	0.9 mg·kg^−1^ at induction	Saline (*n *=* *32) 10.0 ± 3.6 years	MorphineI V	BOLUS: 50 mcg·kg^−1^ in PACU TRIGGER: VAS> 60 mm or OPS >6	POSITIVE* (43%) Ketorolac – 11/32 Control – 25/32 Proportion requiring >1 morphine doses in PACU *Note: data only from ketorolac vs placebo arms*

* Positive, statistically significant opioid dose-sparing effect reported by authors; Negative, no statistically significant difference in opioid requirements.

PR, per rectum; IV, intravenous; IM, intramuscular.

**Table 3:** Pain assessment and Secondary Outcomes

Author (reference no.)	Age	Pain scale (range)	Pain score	Sedation	Respiratory depression	PONV	Pruritis	Urinary retention	Bleeding
Group A									
Hong *et al*. 26	0.5–2 years	CHEOPS 4–13	=	−	0	−	=	X	X
Rugyte *et al*. 18	10–15 years	VAS 0–10	−	=	=	=	X	=	+
Van der Marel *et al*. 17	0–10 months	VAS 0–10	=	X	X	X	X	X	X
Munro *et al*. 19	11–17 years	Numerical 0 – 10	−	X	=	=	=	=	=
Morton *et al*. 20	5–13 years	Numerical 0 – 3	=	=	=	=	X	X	X
Sutters *et al*. 22	Mean 12.6 ± 4 years	Wong-Baker Faces 0–5	−	X	X	=	=	=	0
Sims *et al*. 21	≥7 years (mean 10 years)	VAS 0–10	=	=	X	=	X	X	X
Group B									
Antila *et al*. 25	9–15 years	VAS 0 – 10	−	X	0	=	X	X	=
Oztekin *et al*. 24	5–14 years	Numerical 0 – 10	−	=	0	=	X	X	=
Vetter *et al*. 23	8–16 years	VAS 0–100	−	X	X	=	=	−	0
Group C									
Mireskandari 27	1.5–5 years	CHEOPS	- combined vs placebo	X	X	=	=	X	0
Kokki *et al*. 28	3–16 years	VAS 0–100	=	=	X	=	X	X	=
Maunuksela *et al*. 29	4–12 years	OPS 0 – 9 Numerical 0 – 3	Observer =Patient -	=	X	=	X	X	=
Maunuksela *et al*. 30	1–16 years	Numerical 0 – 9 Numerical 0 – 3	−	=	X	=	X	=	X
Group D									
Hong *et al*. 31	1–5 years	Wong-Baker Faces 0–5	−	−	X	−	=	X	X
Dashti *et al*. 32	7–15 years	VAS 1–100	−	X	0	=	X	X	0
Korpela *et al*. 33	1–7 years	OPS 0–9	X	X	X	=	X	X	=
Sheeran *et al*. 34	>3 years	CHEOPS 4 – 13	=	X	X	X	X	X	0
Viitanen *et al*. 35	1–6 years	OPS 0–9	X	=	X	=	X	=	0
Tuomilehto *et al*. 36	1–9 years	Modified Maunuksela scale 0–10	-IV < placebo	=	X	=	X	=	=
Bremerich *et al*. 37	1–24 months	CHIPPS 0–10	=	X	0	0	X	X	X
Kokki *et al*. 38	1–9 years	Maunuksela 0–10	=	=	X	=	X	=	=
Tuomilehto *et al*. 47	1–9	Maunuksela 0–10	=	=	X	=	X	=	=
Kokki *et al*. 40	1–12 years	Maunuksela 0–10	−	=	X	−	X	X	X
Korpela *et al*. 39	1–11 years	Observer NRS 0–100	−	X	X	−	X	X	X
Kokki *et al*. 41	1–7 years	Maunuksela 0–10	−	=	0	=	X	=	=
Romsing *et al*. 42	5–15 years	Poker Chip Tool 0–4	−	X	X	−	X	X	=
Nikanne *et al*. 43	1–7 years	Maunuksela 0–10	−	−	X	+	X	=	+
Bean-Lijewski *et al*. 44	1–11 years	CHEOPS 4–13	=	=	=	=	X	X	=
Sutters *et al*. 45	7.1 ± 2.4 years	Oucher 0–5 CHEOPS 4–13	−	X	X	X	X	X	=
Watcha *et al*. 46	5–15 years	VAS 0–100 OPS0–9	−	X	X	=	X	X	X

Pain score: ‘=’ no significant difference in control and intervention groups, ‘+’ higher pain score in intervention group compared with control, ‘−’ lower pain scores in intervention group compared with control, ‘and ‘X’ not reported.

Opioid-related adverse effects: ‘=’ equal incidence in both control and intervention groups, ‘+’ higher incidence in intervention group compared with control, ‘−’ lower incidence in intervention group compared with control, ‘0’ indicate absence of this complication in both groups and ‘X’ not reported.

CHEOPS, Children's Hospital of Eastern Ontario Pain Scale; VAS, Visual Analog Scale; OPS, Observer Pain Score; CHIPPS, Children's and Infants Postoperative Pain Scale; NRS, numerical rating scale.

Across the 31 studies, thirty-eight different drug groups were compared with placebo (Figure1). Paracetamol and an NSAID were directly compared within four studies, with three also evaluating the combination of paracetamol and NSAID. Additional within study comparisons included dose-dependent effects of paracetamol 37,39 or ketoprofen 41; different routes of administration of ketoprofen 36,38,47; or administration of ketorolac at the beginning or end of surgery 42. This increased the number of groups in which opioid consumption was measured to 48 treatment arms and 31 placebo controls (one per study) (Table4).

**Table 4:** Summary of study groups and degree of opioid sparing

Study design	Group A	Group B	Group C	Group D
No. of studies	7	3	4	17
No. of active treatment arms	9	3	6	30
Negative arms	paracetamol (×2)		ketoprofen	paracetamol (×4) ketoprofen ketorolac rofecoxib
Positive arms	paracetamol paracetamol + diclofenac diclofenac indomethacin ketoprofen ketorolac (×2)	diclofenac ketoprofen ketorolac	paracetamol paracetamol + diclofenac diclofenac ibuprofen indomethacin	paracetamol (×5) paracetamol + ibuprofen paracetamol + ketorolac ibuprofen ketoprofen (×10) ketorolac (×4) naproxen
% opioid reduction (mean) [95% CI]	**31.6** [16.5–46.6]	**24.3** [−1.7–50.4]	**24.5** [6.3–42.6]	**24.0** [16.4–31.5]

Bold values indicates mean % change in opioid consumption.

### Opioid consumption

**Figure 2:**
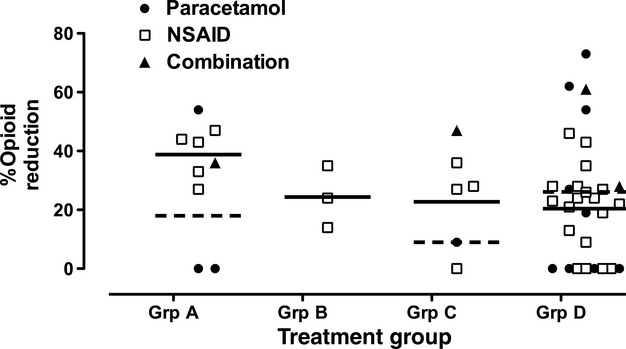
Percentage reduction in opioid requirements in pair-wise comparisons of mean opioid dose requirements in active treatment arms (paracetamol; NSAID; combination = NSAID + paracetamol) vs control/placebo. Studies reporting no statistically significant difference from control are designated as 0% reduction. Solid line = mean of NSAID arms; dotted line = mean of paracetamol arms. Treatment groups comprise Group A = PCA/NCA + study drug ≥24 h; Group B = PCA/NCA + study drug ≤6 h; Group C: intermittent opioid + study drug ≥24 h; Group D = intermittent opioid + study drug ≤6 h.

Significant decreases in opioid consumption by NSAID and/or paracetamol were reported in 21 of the 31 studies, four did not demonstrate any dose-sparing effects, and in six studies, both positive and negative results were reported for different treatment arms (Table1 and 2). Overall, positive effects were reported in 38 of 48 treatment arms (Figure2).

In Group A studies (seven studies with nine treatment arms), surgery was of moderate to major severity, and systemic opioid was available for immediate titration by the patient (PCA) or by nursing staff (NCA bolus plus background). In addition, study drug administration and evaluation continued for at least 24 h following surgery. Seven of nine treatment arms were positive. Two studies reported no significant benefit with addition of rectal paracetamol 17,20. Overall, opioid dose requirements were reduced by 31.6% (95% CI: 16.5–46.6) (Table4). Opioid sparing was reported in all Group B studies, with a mean reduction of 24.3%, but with wide variability, as only three studies were available.

Significant reduction in opioid dose was reported in five of six treatment arms in Group C (mean 24.5% 95% CI 6.3–42.6) and in 23 of the 30 active treatment arms in Group D (mean 24%; 95% CI: 16.4–31.5)(Table4). Overall, the majority of Group C/D studies demonstrated significant opioid sparing, but with much greater variability in reported results.

### Paracetamol vs NSAID vs combination

A higher proportion of positive studies were reported in NSAID (27 of 31; 87%) than in paracetamol (7 of 13; 54%) treatment arms. Four studies included direct comparison of an NSAID and paracetamol. Rectal diclofenac (1 mg·kg^−1^ intraoperative and 8 hrly for 24 h) produced opioid sparing, but by comparison, rectal paracetamol was less effective (40 mg·kg^−1^ intraoperative and 30 mg·kg^−1^ 8 hrly) 27 or showed no benefit (20 mg·kg^−1^ loading and 15 mg·kg^−1^ 6 hrly) 20. Ibuprofen 15 mg·kg^−1^ PR was more effective than paracetamol 40 mg·kg^−1^ PR (27 vs 19% reduction) 35, and preoperative oral administration of naproxen 10 mg·kg^−1^ was more effective than paracetamol 20 mg·kg^−1^ (19% vs nonsignificant difference) 33. Positive opioid sparing by diclofenac (1 mg·kg^−1^ 8 hrly PR) was further enhanced by addition of paracetamol 30 mg·kg^−1^ 8 hrly PR 27, but not by paracetamol 20 mg·kg^−1^ PR loading and 15 mg·kg^−1^ 6 hrly 20. While both paracetamol 40 mg·kg^−1^ PR and ibuprofen 15 mg·kg^−1^ PR reduced opioid requirements following adenoidectomy, combining the two drugs provided no additional benefit 35. A single combined dose of ketorolac and paracetamol markedly reduced postanesthesia care unit (PACU) opioid requirements, but effects of the individual drugs were not assessed 31.

### Within study comparisons: dose response and route of administration

Three studies included dose-response comparisons. Dose-dependent increases in opioid sparing in PACU were reported following 20, 40, and 60 mg·kg^−1^ rectal paracetamol (27 vs 54 vs 73%, respectively) 39. Conversely, rectal paracetamol doses of 10, 20, or 40 mg·kg^−1^ had no significant effect in PACU following cleft palate repair 37. Intravenous ketoprofen 0.3, 1, and 3 mg·kg^−1^ reduced the proportion of children requiring fentanyl for 2 h following adenoidectomy, with minimal dose-related differences (21, 24, and 35% reduction, respectively) 41.

Administration of the same dose of ketoprofen by different routes demonstrated benefit with intravenous but not oral administration 47, and similar degrees of opioid sparing following intravenous vs rectal 38 or intramuscular administration 36. There have been no direct comparisons of paracetamol by different routes. Intravenous 26 but not rectal 17,20 paracetamol reduced 24-h opioid requirements. Wide variability in individual plasma paracetamol concentrations was noted following rectal administration (0.8–59.9 mg·l^−1^) 17.

### Pain assessment and pain scores

All studies incorporated pain assessment, but by a range of different tools. Self-report included numerical rating (0–3 or 0–10), visual analog scales (0–10 or 0–100) or faces scales. Observer tools ranged from an overall numerical rating to composite measures of specific behavioral and physiological responses (e.g., Children's Hospital of Eastern Ontario Pain Scale (CHEOPS) and COMFORT scales) (Table3).

In Group A and B studies, intravenous bolus opioid was triggered by the patient (PCA), who titrated themselves to similar pain scores in two of eight studies 20,21. Six studies reported improved pain scores with addition of NSAID/paracetamol. Outcomes included different composite measures of pain (i.e., area under the pain intensity–time graph for the first 24 h 18, a main effect of treatment with repeated measures anova to 36 h 22, overall pain score for first 12 h 23). Others reported significant reductions in pain score only at some time points (i.e., the first 48 h 19, the initial six postoperative hours 25 or the first hour in PACU 24).

In NCA studies, a background infusion plus opioid bolus administration by a nurse or trained parent following urologic surgery 26, or by a nurse or investigator in intensive care after major abdominal or thoracic surgery 17, resulted in effective titration to similar pain scores. In the latter study, a high proportion of patients were mechanically ventilated, and both Observer VAS and COMFORT scores were low in paracetamol and placebo groups 17.

In Group C and D studies, opioid was available on an ‘as needed’ or ‘PRN’ basis. Nurse administration was triggered by a predetermined pain score using a formal assessment tool (16 studies) or global nursing assessment and patient request (five studies). Pain scores in treatment groups were reported to be significantly lower in the active treatment arm in 10 studies; reduced in some subgroups or on some subscales in 3; equivalent in 6; and were not reported in two studies (Table3).

### Opioid-related adverse effects

Postoperative nausea and vomiting (PONV) was compared in 27 of 31 studies, with six study arms in five studies reporting a significant reduction in the paracetamol and/or NSAID group (Table3). The degree of opioid sparing tended to be greater (47%, 95% CI 22–72) in treatment groups with less PONV when compared to studies with equivalent PONV (26%, 95% CI 20–31). Within study comparisons found a significant reduction in the incidence of PONV with 40 and 60 mg·kg^−1^ PR, but not 20 mg·kg^−1^ which also had less effect on opioid requirement 39. Preoperative, but not postoperative, ketorolac reduced both opioid requirement and the number of children vomiting following tonsillectomy 42.

Ten studies noted no difference in significant adverse respiratory effects, reported as either a lack of respiratory depression or no difference in respiratory rate or episodes of desaturation. Sedation was assessed in 17 of 31 studies: 14 reported no difference, and three studies with positive opioid sparing also reported less sedation in the active treatment group. The incidence of oversedation (defined as Ramsay sedation score >4 on 8-point scale) was reduced when paracetamol 26 or ketorolac plus paracetamol 31 was used with fentanyl NCA; and ‘somnolence’ and IV fentanyl bolus use in PACU was less frequent with addition of ketoprofen 43.

No difference in adverse urinary effects (i.e., need for catheterization or difficulty voiding) was noted in nine studies, but fewer children required a urinary catheter following orthopedic surgery if they received IV ketorolac (1 of 25 vs 7 of 25 in the control group) 23. Six studies recorded pruritus, but there were no differences between treatment and control groups.

### NSAID/paracetamol adverse effects

No cases of accidental overdose or toxicity related to paracetamol were reported.

Alteration in bleeding was the main potential NSAID adverse effect evaluated, particularly as many studies were conducted in children undergoing tonsillectomy. The incidence or degree of perioperative bleeding was reported in 21 NSAID studies: seven each of ketoprofen or ketorolac, two of diclofenac or ibuprofen, and single studies of naproxen or rofecoxib. Bleeding following paracetamol was assessed in three studies, of which two included an NSAID treatment arm. Methods for reporting this outcome varied and included the following: direct measurements of intraoperative blood loss or postoperative blood loss in drains; graded but subjective assessments of intraoperative blood loss by the surgeon; rate of re-operation/interventions to control increased bleeding; or statements that no patients had significant bleeding. Following tonsillectomy and/or adenoidectomy, measured perioperative blood loss was not increased following ketorolac 1 mg·kg^−1^ 42 or ketoprofen 25,28. Graded assessment of blood loss by the surgeon found no increase in intraoperative bleeding with ketorolac 45, ibuprofen 35, or ketoprofen 25,36,38,41. Although not quantified, no cases of increased bleeding were reported following rofecoxib 34, naproxen 33, or paracetamol 32,33. In one study, the rate of ‘more than normal’ bleeding was greater following ketoprofen (12 of 80 vs 3 of 84; *P* = 0.037), but no patients required re-operation 43. Cases of bleeding requiring reoperation were reported in both placebo and/or NSAID groups. Two patients were excluded from analysis following ketorolac as they required an immediate return to the operating theater to control surgical bleeding 42; one patient was withdrawn due to bleeding at 5 h following ketoprofen 18; two patients required diathermy under local anesthesia at 4 or 26 h following ketoprofen 28; and one patient required nasopharyngeal packing overnight following diclofenac 24. Bleeding requiring surgical intervention was also reported in three patients given placebo 24,25,46.

Measured blood loss did not differ from control groups during spinal fusion with ketorolac 19 or ophthalmic, general or orthopedic surgery with ibuprofen 29. No significant episodes of bleeding were reported with diclofenac for cleft palate repair 27, or with ketorolac for orthopedic 23 or day case general surgery 44, despite a greater increase in measured bleeding time (53.4 ± 74.8 s) in the latter study.

### Patient/parent satisfaction

Relatively few studies evaluated overall satisfaction with treatment, and none included patient satisfaction as an outcome. Higher levels of parental satisfaction in the active treatment arm either during the in-hospital stay 26,31 or during both the time in hospital and following discharge 34 were reported. No comparison was made with patient satisfaction, but these studies enrolled infants 26,31 or young children (>3 years; mean 7 years) 34.

## Discussion

Recommendations to use multimodal analgesic therapy for perioperative pain management in children 3,7 are supported by this qualitative systematic review. Across 31 studies, 38 of 48 active treatment arms reported a statistically significant reduction in opioid requirements with co-administration of NSAID and/or paracetamol in pair-wise comparisons with a placebo group. However, potential publication bias against negative studies cannot be excluded. Evidence for a clinical advantage in terms of improved pain scores or a reduction in adverse effects was less robust. However, variability in study design, method of opioid delivery, duration of study drug administration, and reported outcome measures had an impact on the likelihood and degree of positive findings.

### Opioid dose requirements

Opioid-sparing effects with perioperative NSAIDs and paracetamol have been well documented in meta-analyses of adult studies 10,11,48, using a standardized measure of opioid dose (24 h total PCA morphine consumption) 10[additional details in 8]. In the current pediatric series, all studies that evaluated cumulative PCA morphine dose (mcg·kg^−1^·day^−1^ in children aged at least 5 years) reported significant reductions in opioid requirements in the first 24 h by regular doses of NSAID 18–22,25) and in the early postoperative period by a single dose of diclofenac 24 or ketorolac 23. These studies were also included in a meta-analysis of 28 pediatric studies, which calculated the standardized mean difference in opioid requirements for individual trials, and reported significant opioid sparing in PACU and during the first 24 h by NSAID 13. We have also evaluated studies of perioperative paracetamol and found more variable results: rectal paracetamol did not reduce PCA 20 or NCA 17 opioid requirements, but IV paracetamol reduced NCA opioid requirements in children aged 6–24 months 26.

Variable methodology in pediatric analgesic studies influences the sensitivity for detecting differences and the ability to combine data across studies 14,49. Michelet and colleagues 13 also noted significant heterogeneity, but benefit with NSAID was maintained in subgroup analyses of the effects of surgery (adenotonsillectomy vs orthopedic or general surgery) and timing of administration (intra- vs postoperative NSAID) 13. In the current studies, the degree of opioid sparing tended to be higher and more consistent when opioid was readily available for titration (i.e., PCA or NCA) and repeated doses of study drug were given (i.e., Group A design). Studies with opioid available on an intermittent ‘PRN’ basis and evaluating the effect of a single dose of NSAID/paracetamol (i.e., Group D design) also reported significant opioid sparing, but there was much greater variability in the degree of difference and in the outcome being evaluated. Many were conducted following surgery with relatively low analgesic requirements, and group data such as the proportion of patients requiring opioid in PACU, rather than individual dose requirements, were the primary outcomes. Some statistically significant differences may have limited clinical significance (e.g., mean differences of less than one dose per patient). In addition, the duration of follow-up was often limited to time in the PACU or the first 1–2 postoperative hours, with only one study reporting a reduction in analgesic requirements following discharge 39. Reduction in PACU opioid requirements with NSAID has also been confirmed by meta-analysis 13 and while reducing early postoperative pain is clearly important, the greater clinical challenge may be to determine whether this translates into reduced analgesic requirements or improved analgesia following discharge. Recent studies confirm that many children experience significant levels of pain at home 50,51, and provision of adequate analgesia following discharge remains an unmet need.

### Analgesic efficacy

Recruiting children across wide age ranges necessitates use of different measurement tools, and standardized use of validated measures has been advocated 52 to improve comparison across studies. The observer and self-report pain assessment tools used in the reviewed studies have variable numbers of choices and different linear/ratio characteristics, making it difficult to compare absolute changes in pain ‘score’ or intensity or to evaluate an overall change over time. Pain intensity is often not evaluated in adult analyses as it is assumed that patients will titrate themselves to similar levels of analgesia 10,11; but one analysis found pain intensity was reduced at 24 h by multidose NSAID, but not single-dose NSAID or paracetamol 48. In the pediatric meta-analysis, addition of NSAIDs to opioids reduced pain intensity in the PACU but not the first 24 h 13. In 6 of the 8 PCA studies reviewed here, pain scores were lower in the active treatment arms, despite these older children being able to ‘self-titrate’ their analgesia. It is possible children may tolerate higher levels of pain to avoid opioid-related PONV, as has been suggested in adult studies 12. Intermittent opioid administration by a nurse can have less flexible dosing schedules, additional time constraints, and is reliant on the frequency and sensitivity of pain assessment. Nine of 12 Group D studies reported lower pain scores, suggesting that intermittent dosing in the early postoperative period was less effective for titrating analgesia in the placebo groups.

Analgesic trials in children can pose ethical challenges, particularly in the use of placebo control groups 53. Using a rescue-analgesic design with analgesic sparing as a surrogate efficacy endpoint incorporates the scientific and regulatory advantages of placebo-controlled trials, while ensuring children have analgesia available for immediate titration 53. All studies included in this analysis had opioid available for titration postoperatively, either by PCA/NCA or by intermittent bolus. In the majority of studies, both NSAID/paracetamol and placebo treatment arms also received standardized intraoperative opioid and/or local anesthetic infiltration. In some early Group C/D studies, intraoperative analgesia was limited to nitrous oxide alone 29,30,39,44–46. Opioid was available for titration in PACU, and all studies reported significantly higher analgesic requirements in the placebo group 29,30,39,44–46. Differences in intraoperative analgesia may also contribute to a cross-study variability in opioid sparing, particularly when assessment is limited to the first few postoperative hours.

### Opioid-related adverse effects

Although statistically significant reductions in opioid requirements demonstrate analgesic benefit, clinical benefit is enhanced if there is also a reduction in opioid-related adverse effects. Individual studies are rarely powered for these secondary outcomes and definitions or thresholds for reporting adverse effects vary across studies. Postoperative vomiting is an important cause of morbidity, a leading concern for parents and patients, and may require readmission 54,55. In the first 24 postoperative hours, NSAIDs had a similar impact on PONV in meta-analyses of adult (odds ratio 0.7, 95% CI 0.53–0.88) 10 and pediatric (odds ratio 0.75, 95% CI 0.57–0.99) 13 studies. In addition to patient (i.e., age, gender) and anesthetic factors, the type of surgery can have a significant effect. Many pediatric studies have been conducted following tonsillectomy, which has a high rate of PONV, and NSAIDs had a greater impact in this subgroup 13. There have been insufficient studies to specifically evaluate PONV in other high-risk surgical groups, such as strabismus, although beneficial effects of NSAID on both opioid sparing and vomiting have been reported 40. Although some studies evaluated the number of episodes of vomiting in individual children 28,40, the majority of studies reported the incidence of vomiting within treatment arms, and it was not possible to differentiate effects on the frequency or severity of vomiting. Other opioid-related adverse effects, such as urinary retention and pruritus are less common, are less likely to be reported in individual studies, and no significant differences were reported in a meta-analysis 13.

Respiratory depression is the most feared adverse effect of opioids, with an incidence in large pediatric audits (>10 000 patients) of 0.13% with opioid via continuous infusion, PCA, or NCA 1 and 0.4% with opioid NCA in a younger population 2. Clinical trials are not powered to evaluate this rare outcome and often exclude patients shown to be at highest risk (i.e., neonates, particularly those born preterm, and patients with comorbid conditions such as cardiorespiratory disease and neurodevelopmental impairment) 1,2. Increased sedation, which can be a more reliable indicator of impending respiratory depression, was noted in some placebo groups, but was not sufficient to be associated with respiratory depression or oxygen desaturation. 26,31,43.

### Type of surgery

Studies included here and in previous analyses of perioperative opioid dose requirements in children 7,13 include patients undergoing a range of different surgeries, with variable perioperative analgesic requirements. When opioid requirements were relatively high and administration via NCA or PCA was required, results were less variable and the ability to detect significant differences with co-administration of NSAIDs was enhanced. NSAIDs may have specific efficacy against bone pain, and one within study comparison showed benefit with ibuprofen following orthopedic but not general surgery 29. Tonsillectomy not only has an impact on potential opioid-related adverse effects such as PONV, but analgesic benefits must also be weighed against the potential for NSAID-induced bleeding. Cases of post-tonsillectomy bleeding were noted in both NSAID and placebo groups but the number requiring intervention was small. Meta-analyses of pediatric studies have concluded that the risk of bleeding requiring reoperation is not increased by diclofenac for acute pain 56 or NSAIDs following tonsillectomy 57.

### Comparison of study drugs

There is currently insufficient data to determine the relative efficacy of paracetamol or different NSAIDs, and the dose equivalence of different preparations at different ages is not well-established. Overall, the proportion of positive studies was lower with paracetamol, and adult analyses also suggest a greater degree of opioid sparing with NSAIDs vs paracetamol 10. However, pediatric studies may also be confounded by inadequate paracetamol dosing and variability in absorption, particularly when given by the rectal route 17. Within study comparisons found dose-dependent increases in opioid sparing with higher rectal paracetamol dose 37,39. The time to peak plasma concentration varies with the rectal preparation, but can exceed 2 h 58,59. In addition, the equilibration half-life (t_eq_) for the analgesic effect compartment is over 50 min, which further delays time to maximum analgesia 60. As a result, an adequate effect site concentration may not be achieved following administration of single doses for relatively brief surgical procedures with evaluation in PACU; a design commonly utilized in the studies classified here as Group D, which show wide variability in reported opioid sparing and the greatest number of negative paracetamol studies. Intravenous paracetamol was beneficial in 2 included studies 26,31 and has recently been reported to reduce opioid requirements in neonates and infants following major surgery 61. Recent pharmacokinetic analyses provide further data regarding appropriate dose schedules for intravenous paracetamol 62,63.

Combining an NSAID and paracetamol produced variable benefit in individual studies. Using time-effect profiles at different doses, a recent simulation with paracetamol and ibuprofen suggests analgesic benefit with this combination is more likely to be seen with modest doses of drug and at time points beyond PACU (i.e., >2 h) 64. High doses of NSAID approach a maximum or ceiling effect, and little additional benefit may be gained by adding paracetamol 64. Additive analgesia with paracetamol and NSAID has been demonstrated in adults with acute pain 9,16,65, and although co-administration did not significantly alter opioid requirements, meta-analysis of pediatric studies reported a further reduction in pain intensity during the first 24 h when regular paracetamol was added to NSAID 13.

### Limitations and future directions

The current systematic review is limited to a qualitative analysis; however, findings are consistent with a recent meta-analysis which evaluated the impact of NSAID on postoperative opioid requirements in children 13. This meta-analysis provides more detailed quantification of the degree (i.e., standardized mean difference) in opioid requirement, and by combining data for secondary outcomes, such as PONV, can better evaluate effects for which individual studies are inadequately powered. However, significant heterogeneity is often seen in pediatric analgesic studies, with variability in design, study population, and outcome measures. Qualitative reviews can provide further information about which clinical populations are most likely to benefit from the intervention and highlight areas requiring further research. There is an ongoing need for more uniform use of validated pain scores and outcome measures 52 to facilitate comparison and combination of data from different trials. Standardized definitions and reporting of adverse effects or clinical endpoints such as PONV, pruritus, and sedation would also enhance evaluation of relative risks and benefits. Further quantification of clinically significant benefits is likely to require much larger or multi-center studies that are sufficiently powered to detect differences in adverse effects rather than just differences in opioid consumption 10 and/or that have more prolonged follow-up that includes evaluation of pain and function following discharge after short-stay or day case surgery.

## Conclusion

NSAIDs and/or paracetamol reduce perioperative opioid requirements in children, and positive effects are most consistently seen when opioid requirements are relatively high and titrated by NCA or PCA. The degree of clinically significant benefit in terms of improved analgesic benefit or reduction in opioid-related adverse effects varies across studies, and there is currently insufficient data to compare the relative efficacy of different drugs. The doses of NSAIDs and/or paracetamol utilized in these trials were not associated with any additional adverse effects. These data provide further support for use of multimodal analgesia for perioperative pain in children.
